# Parentage Analysis in a Green Sea Turtle (*Chelonia mydas*) Population From French Polynesia Reveals a Tendency for Inbreeding and Unexpected Plasticity in Reproductive Behaviour

**DOI:** 10.1002/ece3.70855

**Published:** 2025-07-16

**Authors:** Violaine Dolfo, Cécile Gaspar, Miri Tatarata, Emilie Boissin, Serge Planes

**Affiliations:** ^1^ PSL Research University: EPHE–UPVD–CNRS UAR 3278 CRIOBE Perpignan France; ^2^ Te Mana O Te Moana Foundation Faaa French Polynesia; ^3^ Direction de l'Environnement de la Polynésie Française Papeete French Polynesia; ^4^ Laboratoire d'Excellence « CORAIL » Perpignan France

**Keywords:** genotype reconstruction, inbreeding, mating behaviour, multiple paternity, relatedness, reproductive success

## Abstract

The reproductive systems of natural populations can significantly impact their genetic diversity by either preventing or promoting inbreeding. Therefore, it is crucial to have a comprehensive understanding of the mating system to evaluate a population's ability to maintain genetic diversity over time. In this study, we examine the mating system of an endangered population of green sea turtles in Tetiaroa, French Polynesia. We determine if different mating behaviours serve as strategies to avoid inbreeding. We genotyped 107 nesting females and 1483 hatchlings from 549 nests and used 23 microsatellite markers to reconstruct the genotypes of the sires. We assessed the level of inbreeding and relatedness among the parent pairs and explored the correlation between relatedness and indicators of reproductive success. We investigated the mating behaviours of both males and females and determined whether specific behaviours were linked to different levels of relatedness. We explored for the first time the relatedness bias of mating behaviours in green turtles. Our results showed that the global *F*_*is*_ was significant in the population, and the levels of relatedness were higher than expected through random mating, indicating inbreeding and non‐random partner selection for related mates. No mating behaviours were associated with lower relatedness levels, suggesting inbreeding tolerance or preference in this population. Finally, we discovered unexpected plasticity in the reproductive frequency of females, the length of the inter‐nesting interval, and the relative timing of breeding and nesting. If confirmed in other populations and with a larger sample size, these new findings may reshape our understanding of the green turtle's intricate reproductive system.

## Graphical abstract

In this study, we examine the mating system of an endangered population of green sea turtles in Tetiaroa, French Polynesia, through genotype reconstruction and parentage analysis. We unravelled the mating behaviours of both males and females and determined if they serve as strategies to avoid inbreeding by investigating levels of relatedness between mating partners. Levels of relatedness were higher than expected through random mating, indicating non‐random partner selection and no behaviour was significantly associated with an inbreeding avoidance strategy.

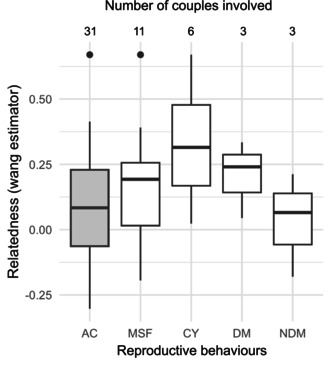

## RESUME

Les systèmes de reproduction des populations naturelles peuvent avoir un impact significatif sur leur diversité génétique en empêchant ou en favorisant la consanguinité. Il est donc essentiel d'avoir une compréhension globale de ces systèmes pour évaluer la capacité d'une population à maintenir la diversité génétique au fil du temps. Dans cette étude, nous examinons le système de reproduction d'une population menacée de tortues vertes à Tetiaroa, en Polynésie française. Nous déterminons si différents comportements de reproduction servent de stratégies pour éviter la consanguinité. Nous avons génotypé 107 femelles pondeuses et 1483 nouveau‐nés provenant de 549 nids, et utilisé 23 marqueurs microsatellites pour reconstruire les génotypes des pères. Nous avons évalué le niveau de consanguinité et de parenté entre les paires de parents et exploré la corrélation entre la parenté et les indicateurs de succès de la reproduction. Nous avons étudié les comportements de reproduction des mâles et des femelles et déterminé si des comportements spécifiques étaient liés à différents niveaux de similarité génétique. Nos résultats ont montré que le *F*_is_ global était significatif dans la population et que les niveaux de parenté étaient plus élevés que dans un scénario d'accouplements aléatoires, indiquant une consanguinité et une sélection non aléatoire de partenaires. Aucun comportement d'accouplement n'a été associé à des niveaux de similarité génétique inférieurs à ceux de la population générale, suggérant une tolérance ou une préférence de consanguinité dans cette population. Enfin, nous avons découvert une plasticité inattendue dans la fréquence de reproduction des femelles, la durée de l'intervalle entre les nidifications et entre la reproduction et la nidification. Si elles sont confirmées dans d'autres populations et avec un échantillon plus large, ces nouvelles découvertes pourraient remodeler notre compréhension du système reproducteur complexe de la tortue verte.

## 1 Introduction

Reproductive systems strongly influence genetic diversity in natural populations, and subsequently, the long‐term viability of populations (Booy et al. 2000). Loss of genetic diversity may lead to inbreeding depression, loss of adaptive potential, and accumulation of deleterious alleles, and ultimately to extinction (Charlesworth 2009). The number of breeders, their reproductive success and their mating strategies are key elements of the mating system of a population (Sugg and Chesser 1994). A good understanding of these factors is essential to assess the intrinsic capacity of natural populations to maintain their genetic diversity through generations (Anthony and Blumstein 2000). Several components of the mating system may either mitigate or favour inbreeding. They may be pre‐copulatory such as the operational sex ratio (OSR, i.e., the relative number of available breeders from both sexes, (Emlen and Oring 1977)), reproductive frequency, and partner choice and number (Blouin and Blouin 1988; Taylor, Price, and Wedell 2014). Post‐copulatory factors include sperm storage and sperm competition (Michalczyk et al. 2011).

OSR is a key feature of the mating system, especially for species with a temperature‐dependent sex determination (TSD) like marine turtles (Standora and Spotila 1985). The primary sex ratio of TSD species is solely dependent on environmental conditions and can be heavily biased under climate warming situations (Janzen 1994). The primary sex ratio of several populations of marine turtles is female‐biased (Casale, Gerosa, and Yerli 2000; Jensen et al. 2018; Santidrián Tomillo et al. 2014; Zbinden et al. 2007), but the OSR is usually much more balanced (Hays, Shimada, and Schofield 2022), mitigating concerns about the vulnerability of a population. However, as highlighted by Wright, Fuller, et al. (2012), if the OSR reflects a small number of males that breed more frequently than females, it will still lead to a loss of genetic diversity and inbreeding. Due to the high energetic cost of reproduction, which may include long migrations between foraging and nesting grounds, female marine turtles typically reproduce at intervals of several years (Hays, Mazaris, and Schofield 2014). For the green turtle, *Chelonia mydas* , the reproductive frequency in females is estimated between 2 and 5 years (Seminoff et al. 2015). In contrast, males can mate more frequently, and annual migrations to breeding grounds have been observed in some leatherback turtles foraging off Canada ( *Dermochelys coriacea* ) (James, Eckert, and Myers 2005), and some green turtle individuals in Queensland (Australia) (Limpus 1993). In another population of green turtles in Cyprus however, reconstruction of paternal genotypes revealed that males do not breed annually (Wright, Fuller, et al. 2012). Thus, knowing the reproduction frequency of both sexes in a population is important in order to correctly interpret the OSR and better estimate the inbreeding risk.

Both male and female marine turtles can reproduce with several partners (i.e., polygynandry), which tends to equilibrate the OSR (Jensen, FitzSimmons, and Dutton 2013). Polyandry is observed through multiple paternity in clutches, which has been found at various degrees in all marine turtle species. For green turtles, it ranges from 15% to 92% of the clutches depending on the populations (reviewed in Lee et al. 2018). Multiple paternity is a direct consequence of seasonal sperm storage in females, a capacity well established in Testudines (Pearse and Avise 2001) and observed in six of the seven marine turtle species (Crim et al. 2002; Fitzsimmons 1998; Kichler et al. 1999; Phillips et al. 2013; Sakaoka et al. 2011; Theissinger et al. 2009). Satellite tracking of males and females and direct observations of mating pairs showed that mating usually immediately precedes the nesting season (Hays, Shimada, and Schofield 2022) and sperm storage allows subsequent multiple nesting (Lasala, Hughes, and Wyneken 2020). However, sperm storage across multiple seasons has not been formally demonstrated in marine turtles, as opposed to terrestrial and freshwater turtles (Owens 1980; Whitaker 2006). Several studies have suggested that it is likely (Howe et al. 2017; Theissinger et al. 2009; Wright et al. 2013), but it has not occurred in other studies (Sakaoka et al. 2013).

It has been proposed that females would benefit from polyandry and sperm storage. The ‘good genes’ hypothesis relies on the assumption that by mating with different partners, better quality sperm would outcompete lower quality sperm, which would lead to increased fitness of embryos sired by the dominant sire (Kokko et al. 2002). However, while many studies have attempted to prove this hypothesis, no correlation has yet been found between multiple paternity and fitness parameters in the clutches (Jensen et al. 2006; Lee et al. 2018; Lee and Hays 2004; Wright et al. 2013, but see Howe et al. 2017). On the other hand, the ‘genetic compatibility’ hypothesis predicts that paternity would be biased towards genetically dissimilar males to avoid inbreeding (Bretman, Newcombe, and Tregenza 2009; Zeh and Zeh 1997). In an attempt to test this latter hypothesis in a hawksbill turtle population in the Republic of Seychelles, Phillips et al. (2013) did not find any correlation between relatedness and paternity contribution in the clutches. Ultimately, all of these studies tend to conclude that polyandry is likely to occur as an energy cost trade‐off between mating several times and avoiding mating harassment (i.e., convenience polyandry, Lee and Hays 2004). Male sea turtles are known to actively and aggressively attempt mating, and females try to avoid it (Booth and Peters 1972; Reina et al. 2005). Thus, levels of multiple paternity may simply reflect the density of breeders on reproductive grounds and the number of encounters between the two sexes (Lee et al. 2018).

Understanding the mating system of sea turtles is challenging, notably due to the limited observations of males in general, and especially during breeding periods. For species as elusive as these, molecular analyses can provide important insights. The use of microsatellite markers allowed for the genotypes of unsampled sires to be reconstructed and to thus assess the mating system parameters in several marine turtle populations (e.g., Bernatchez and Duchesne 2000; Figgener et al. 2016; Horne et al. 2022; Phillips et al. 2013; Wright, Stokes, et al. 2012). Together, these studies reveal that regional variations in these parameters are the rule rather than the exception, and many regions are still lacking this key information for their populations.

One such region of interest is French Polynesia, an archipelago with significant nesting grounds for green turtles. French Polynesia archipelago is composed of 118 islands distributed over an exclusive economic zone of 5 million km^2^, a surface as wide as Europe. The country was thought to have hosted approximately 1000 female green turtle breeders in the early 1990s (Groombridge and Luxmoore 1989), although the population has likely declined due to continuous threat, and no recent assessment exists (Allen, Martin, and Jones 2023; Seminoff et al. 2015). In the centre of French Polynesia, Tetiaroa Atoll (Society archipelago) is one of the major nesting grounds. It is estimated that around 100 females nest annually on the atoll (Seminoff et al. 2015; Touron, Genet, and Gaspar 2018), and mating behaviours are observed around the island (Gaspar, pers. comm.). This location provides a unique opportunity to closely investigate the mating system of green turtles with molecular marker analyses. Green turtles are globally endangered (Seminoff 2023) and populations in French Polynesia are genetically isolated from other populations in the Pacific Ocean (Dolfo, Gaspar, et al. 2023). Understanding the reproductive strategies of these turtles would offer valuable insights into their risk of genetic diversity loss, which is an important indicator for long‐term conservation plans.

Thus, the aim of this study is threefold. First, we characterise the level of inbreeding and relatedness in the population of green turtles nesting in Tetiaroa and explore their correlation with two indicators of reproductive success in the clutches: the fertilisation success, defined as the ratio between fertilised eggs and total clutch size, and the hatching success, defined as the ratio between successful hatchlings and fertilised eggs. Second, using reconstructed pedigrees, we determine the features of the mating system and nesting parameters of this population. These parameters, which are key to inbreeding depression dynamics, include the breeding sex ratio, the reproductive frequency, the level of multiple paternity, the female's nesting intervals, and the number of partners for the males. Third, we test whether the mating strategies deployed reduce the overall relatedness and contribute to avoiding inbreeding by comparing the relatedness of couples involved in these strategies with the overall relatedness of the population. To our knowledge, this work is the first to explore the relatedness bias of mating behaviours in green turtles. It improves our general understanding of the drivers of mating strategies in sea turtles.

## 2 Material and Methods

### 2.1 Study Site and Sample Collection

**FIGURE 1:**
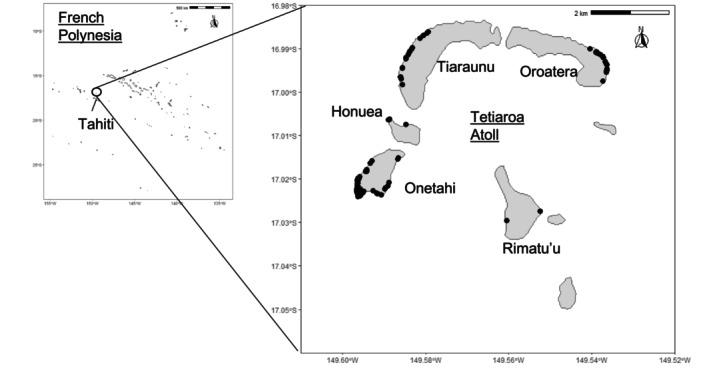
Sampling locations on Tetiaroa Atoll, French Polynesia. Sampled nests are represented with black dots around the islets.

Sampling was conducted on Tetiaroa atoll (16°59′ S, 149°34′ E), French Polynesia, during 11 nesting seasons from 2010/11 to 2020/21. Sample collection was authorised and coordinated by the Department of the Environment of French Polynesia (DIREN). Tetiaroa atoll has a total surface of 6 sq. km, about 585 ha of sand, and is divided into 12 islets (Figure 1). Between 2010/11 and 2017/18, between 53 and 1316 nesting events per season (July to April) were recorded by the local NGO *Te mana o te moana* (Touron, Genet, and Gaspar 2018). Biopsies of approximately 0.5 cm^3^ of skin and muscle tissues were collected from the posterior hind limb of all observed females and all dead hatchlings and embryos. For a limited number of nests, sampling of the live hatchlings was allowed by the DIREN, resulting in a few nests having more than 10 sampled hatchlings (Data S1). The laying date was either recorded when laying was directly observed, or estimated when the nest was discovered. In this case, a confidence interval of ±3 days was applied to all estimations. Since 2010/11, monitoring has gradually increased and by 2016/17, almost all nests were sampled each season. However, nesting females were not always observed (Touron, Genet, and Gaspar 2018). Nest parameters such as clutch size, number of hatchlings (estimated from empty eggshells), number of dead embryos, and number of unfertilised eggs were recorded for each nest. The error rate for estimating hatchling numbers from empty eggshells is assumed to be negligible for this species (Ceriani et al. 2021). Samples were stored in 90% ethanol and kept at 4°C or −20°C until processing.

### 2.2 Molecular Analyses

Total genomic DNA was extracted using the QIAamp 96 DNA QIAcube HT Kit and the QIAcube DNA extraction robot (QIAGEN, Hilden, Germany) following the manufacturer's protocol with modification as described in Dolfo, Boissin, et al. (2023). Samples were genotyped at 23 microsatellite loci using five multiplex reactions as per Dolfo, Boissin, et al. (2023) (GenBank accession number: OQ162049‐OQ162073). Allele sizes were visually assessed using GENEMAPPER software v.5 (Applied Biosystems). All ambiguous peak profiles were considered as missing data and only individuals with missing data which occurred at 3 loci or less were kept for the analyses. MICROCHECKER v.2.2.3 (Van Oosterhout et al. 2004) was used to identify null alleles, stuttering errors, and allele dropout. The genotyping error rate was calculated with COLONY v.2.0.6.6 (Jones and Wang 2010) (see paragraph Calibration of the set of loci below).

### 2.3 Conceptual Workflow

**FIGURE 2:**
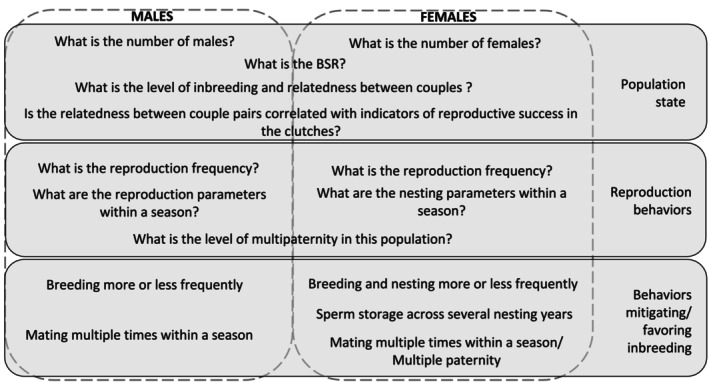
Conceptual workflow for all the analyses performed for this study.

To answer the three goals defined above, analyses were carried out with the following conceptual workflow (Figure 2). First, the population state was described through the number of breeders, the breeding sex ratio, and the level of inbreeding and relatedness between reconstructed couples. While the true OSR cannot be calculated through genetic inferences because not all available breeders can be accounted for, we used the breeding sex ratio (BSR, the successful number of mating individuals) as a proxy for this parameter (Stewart and Dutton 2014). The effect of the relatedness on the indicators of reproductive success in the clutches was investigated (i.e., fertilisation success and hatching success). Then, the reproductive behaviours of the population were described for both sexes, that is, the reproduction frequency, nesting and/or reproductive parameters within a season and levels of multiple paternity. Finally, we tested whether these behaviours mitigate or favour inbreeding by comparing the relatedness of couples involved with the relatedness of all reconstructed couples.

### 2.4 Identification of Recaptured Individuals

The R package *RClone* (Bailleul, Stoeckel, and Arnaud‐Haond 2016) was used to identify duplicate genotypes in the dataset and to detect recaptured individuals. Using the allele frequencies of the dataset, *RClone* simulates a reproduction event and compares the genetic distances between genotypes in the simulated and the observed populations. This allows for a threshold to be determined, below which genotypes in the observed population may be too similar to occur as a result of reproduction. Thus, the genotypes of all individual pairs that had < 5 differences at any locus were double‐checked with GENEMAPPER, and the data were crosschecked with field information to identify recaptured individuals.

### 2.5 Calibration of the Set of Loci

To test the accuracy of the motherhood assignment provided by the software COLONY v.2.0.6.6 (Jones and Wang 2010) and in order to minimise type II errors (i.e., retaining incorrect mother‐offspring assignments), we calibrated the assignment probability based on known mother‐offspring relationships from the field. This involved 58 dams and 275 hatchlings from 105 nests. We determined the appropriate probability threshold in our population by comparing the probability associated with correct mother‐offspring assignments (based on field data) vs. incorrect assignments (see Data S2). COLONY uses a full likelihood model to assign sibship and parentage relationships using multilocus genotype data. Its algorithm allows for missing data and genotyping errors. Three long runs were performed with high likelihood precision, allowing polygamy for both sexes under an inbreeding model, and assuming an error rate of 0.01 for both allelic dropout and genotyping error. It is assumed that hatchlings sampled in 2010/11 did not reach sexual maturity by 2020/21 based on the mean age at maturity of 25 years (Chaloupka, Limpus, and Miller 2004). Therefore, we did not consider the occurrence of overlapping generations. All the hatchlings were considered as potential offspring and all the females were candidate mothers (dams).

Genotyping errors for all 23 loci were calculated by COLONY (Data S2). To determine the effect of genotyping errors on the accuracy of motherhood assignment, the above calibration was run with three different subsets of markers to determine which one would best perform in assigning true mother‐offspring relationships: subset 1 comprised all 23 loci described in Dolfo, Boissin, et al. (2023); in subset 2 the loci which were deviant from Hardy–Weinberg equilibrium, as emphasised by the authors, were removed, leaving 14 loci (list in Data S2); in subset 3 the loci which were given the highest error rate by COLONY were removed (CMY19, CMY22 and CMY32), leaving 20 loci. Based on this calibration (Data S2) all 23 loci were retained for the analyses.

### 2.6 Parentage Analyses

COLONY was run with 23 loci in the same configuration as for marker set calibration, to assign candidate mother‐offspring relationships and to infer the genotype of unknown parents. For genotyped dams, an offspring assignment probability threshold of 0.9 was used based on the results of the above calibration (Data S2). For genotypes that were reconstructed, COLONY only provides a probability per locus. A probability threshold of 0.9 is usually a good choice for conservative results; however, in our case, this threshold would discard 93% of the genotypes. Thus, probability thresholds from 0.4 to 0.9 were examined to determine the best trade‐off between conservative and robust results (i.e., number of samples retained) (Data S3). Finally, only reconstructed loci with a probability superior to 0.8 were retained for the analyses, while the others were considered as missing data. Like genotyped individuals, individuals with missing data at more than 3 loci were discarded.

**FIGURE 3:**
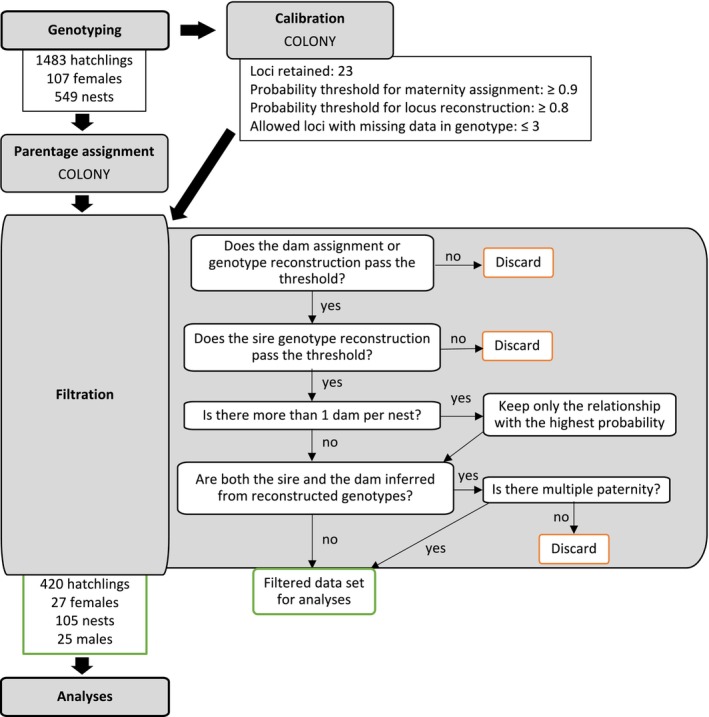
Workflow demonstrating the sequential steps for processing genotype data, the decision tree utilised to filter out parentage assignments, and the number of individuals retained after genotyping and filtration.

Finally, the dataset was checked for inconsistencies (e.g., when more than one dam was found for a single nest) and the least likely relationship (i.e., with the lowest probability) was removed. When both the dam and the sire were inferred from reconstructed genotypes, it is impossible to know which genotype truly corresponds to the dam, and which corresponds to the sire (they could be interchanged without consequences on the offspring genotype). Thus, unless multiple paternity was detected, these configurations were also removed from the data set. Ultimately, a parent‐offspring relationship was only retained when both parents (assigned or reconstructed) passed the different thresholds: 0.9 for assigned dams, 0.8 for reconstructed genotypes and < 3 missing loci for a given genotype (Figure 3).

From parentage assignments, the breeding sex ratio (BSR), and female and male reproduction frequencies were determined. Additionally, within‐season parameters such as multiple paternity, female nesting interval, and the number of partners for males, were reported. Multiple paternity was investigated only in the nests counting a minimum of 3 genotyped hatchlings. The levels of fertilisation success (fertilised eggs/total clutch size) and hatching success (successful hatchlings/fertilised eggs) were compared in clutches with and without multiple paternity using a non‐parametric Wilcoxon rank sum test on the median (after testing for the normality of the distribution with the Shapiro–Wilk test).

### 2.7 Relatedness and Inbreeding

The inbreeding coefficient (*F*_*is*_) was calculated with GENETIX v4.05.2 (Belkhir et al. 2004) on all genotyped females and one offspring per nest (*n* = 665). Pairwise relatedness coefficients (*r*) were calculated with the R package *related* v1.0. (Pew et al. 2015) for all parent pairs found using COLONY. Simulations were performed based on the observed allele frequency to determine which of the four relatedness estimators implemented in the package performs best on our data (i.e., is the closest to expected relatedness values). A hundred simulated genotypes for each of the four relationships were generated and their *r* coefficient was calculated (parent‐offspring, expected *r* = 0.5; full‐sibling, expected *r* = 0.5, half‐sibling, expected *r* = 0.25, unrelated, expected *r* = 0). The Wang estimator, which yielded the best fit to expected relatedness values, was then used for relatedness calculation of reconstructed parent pairs (Data S4). Relatedness of reconstructed vs. potential parent pairs was calculated, and a Wilcoxon rank sum test was performed to test for significant differences between the two relatedness distributions (the normality of distributions was rejected with the Shapiro–Wilk test). To explore the effect of relatedness on reproductive success, fertilisation and hatching success were plotted against the relatedness of parent pairs. Additionally, the relatedness of pairs involving (i) a male that mated with multiple partners (within a season or across seasons), (ii) a female that mated with multiple partners (within a season or across seasons), (iii) the female and dominant male from clutches with multiple paternity and (iv) a female that nested over several years, were compared with the relatedness of all the reconstructed parent pairs to determine whether any of these behaviours led to a deviation of the observed relatedness, and significant differences on the median relatedness were tested with a Wilcoxon rank sum test.

## 3 Results

### 3.1 Recapture Identification

Genotypes were obtained from 1483 hatchlings representing 549 nests and 107 females. Sample size per nest ranged from 1 to 83, with 15 nests having more than 10 samples (Data S1). The 23 loci had a mean *H*_*e*_ of 0.66 ± 0.04 and a mean *H*_*o*_ of 0.62 ± 0.04. Using the R package *RClone*, two genotype clones were identified (Table 1). They correspond to two females; both were sampled for the first time in 2016–2017 and resampled after 4 years in 2020–2021. One of these females was correctly identified in the field by its flipper tag, while the other was thought to be an unknown female and was given a different name at the second encounter.

**TABLE 1:** Females found nesting in multiple seasons using *Rclone* analysis (marked with°) and parentage analysis (marked with ^+^).

Ind #	Ind. ID	First sampling season	Second encounter	First genotype reassignment	Second genotype reassignment
1	CMY1113°^,+^	2016/17	2020/21	2017/18	—
2	CMY1139°	2016/17	2020/21^a^	—	—
3	CMY1104^+^	2016/17	—	2017/18	—
4	CMY1105^+^	2016/17	—	2017/18	—
5	CMY1101^+^	2016/17	—	2015/16	2017/18

^a^ Different ID was given on the second encounter.

### 3.2 Parentage Analysis

After COLONY's parentage reassignment, genotype reconstruction, and filtering with the different thresholds, 105 nests were assigned to 27 genotyped dams and accounted for 420 offspring between seasons 2014/15 and 2020/21 (Data S5). One hatchling that was not assigned to any nest was linked with a genotyped dam. Of these 105 nests, 37 had more than 2 genotyped hatchlings, and 6 contained more than 10 hatchlings. Once the sires' genotypes were reconstructed, we identified a total of 25 sires that explained the genotypes of the 420 hatchlings (Data S5).

### 3.3 Inbreeding and Relatedness in the Population

**FIGURE 4:**
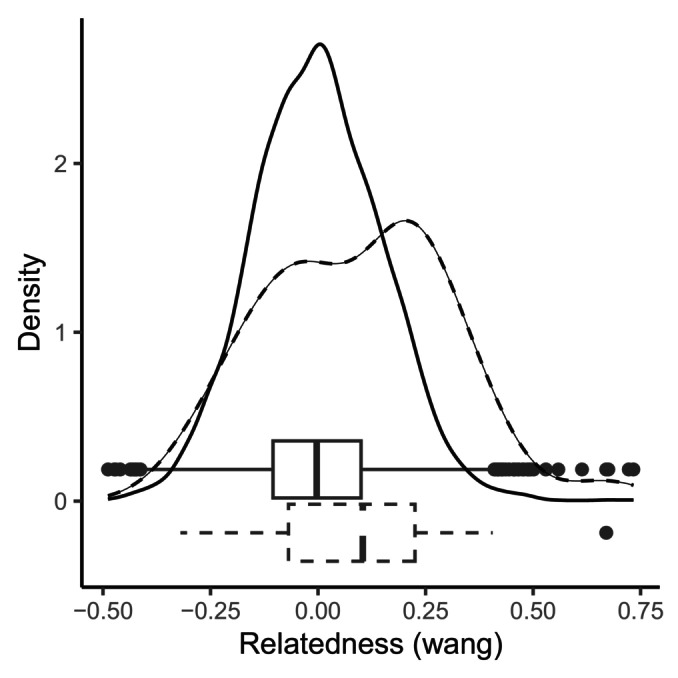
Density and boxplot of the relatedness of all the potential (full line) and reconstructed (dash line) parent pairs.

**FIGURE 5:**
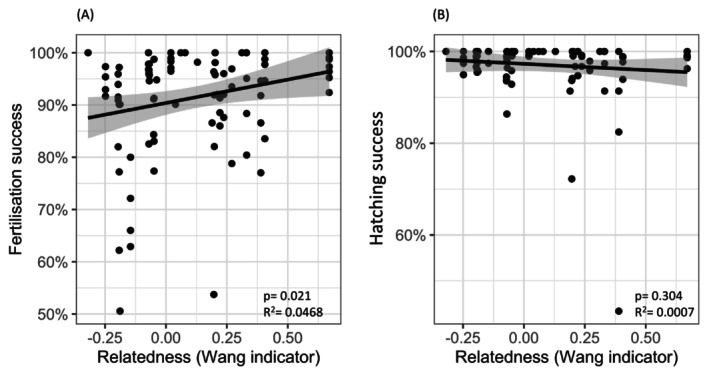
Correlation between the relatedness of parent pairs and two indicators of reproductive success in the clutches: (A) fertilisation success, and (B) hatching success. Linear regression with a 95% confidence interval (grey area) is represented. *p*‐values and *R* ^2^ for a linear model are indicated.

Global *F*_is_ was significant (*F*_is_ = 0.06, *p*_value < 0.001) showing inbreeding in the population. Pairwise relatedness was calculated for all possible male–female dyads and reconstructed couples. The median relatedness of reconstructed couples (median = 0.084) was higher than the median relatedness of all possible dyads (median = −0.007) (Figure 4) and this difference was significant (non‐parametric Wilcoxon rank sum test, *p* = 0.008). The percentage of pairs with a relatedness > 0.25 and 0.5 was also higher for the reconstructed couples than for the potential pairs (*r*_0.25_: 19% vs. 4%, *r*_0.5_: 3% vs. 0.18%, Table 3). When looking at the effect of the relatedness of the parents on the indicators of reproductive success in the clutches, a small positive correlation was found with the fertilisation success (*p* = 0.021, *R* ^2^ = 0.0468), and no correlation was found with the hatching success (*p* = 0.304, *R* ^2^ = 0.0007) (Figure 5).

### 3.4 Frequency of Reproduction and Within‐Season Nesting Parameters

Out of the 27 females, 23 of them nested for only one season. The remaining four females were observed nesting across consecutive seasons. Among these, three had offspring in two seasons and one had offspring for three seasons, indicating an annual reproduction cycle for these females (Table 1). Each of them laid between 3 and 10 clutches sired by a unique male over the period. The interval between the first and last clutch was 421–674 days. For these females, we did not find any other clutch sired by other males within this period.

**FIGURE 6:**
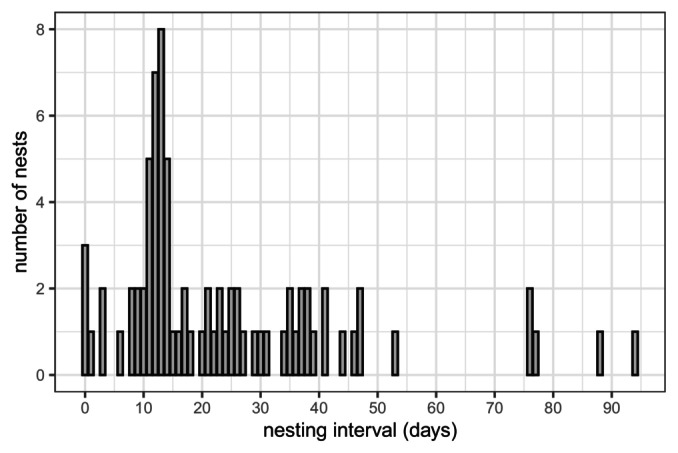
Inter‐nesting interval between two successive nesting events for every female between 2014/15 and 2020/21 in Tetiaroa atoll.

In a single season, females laid between 1 and 10 clutches (Data S6), with an average of 3.2 nests per female. The most common nesting interval was 11–14 days, with variations ranging from 0 to 94 days (Figure 6). Six clutches followed a first egg‐laying event by only 0–3 days (±3 days), indicating that a single female can lay several clutches in a very short time interval, possibly on the same day. The size of these clutches varied between 39 and 103 eggs (mean 79 eggs), which was not different from the mean clutch size of the total sample (84 eggs, *t*‐test *p*‐value = 0.43 with normal distribution) (Data S7).

Of the 25 reconstructed males, 19 were assigned offspring in only one season, 5 had offspring in 2 seasons (4 in consecutive seasons and 1 with a gap period of 3 years), and 1 male had offspring in 3 consecutive seasons. Among the six males found in different seasons, four were associated with a different female on the second encounter. Over the sampling period, only three males mated with several females within a single season. The breeding sex ratio ranged from 0.88 to 1.33 males per female depending on the season, averaging 1.02 over the period, but the sample size was small with < 10 males and females per season (Table 2). Thus, temporal variations between the nesting seasons were not investigated.

**TABLE 2:** Number of females and males deduced from parentage analysis for each season, and breeding sex ratio (BSR, number of males for one female).

Season	Nb. females	Nb. males	BSR
2014/15	3	4	1.33
2015/16	2	2	1.00
2016/17	8	7	0.88
2017/18	5	5	1.00
2018/19	3	3	1.00
2019/20	5	4	0.80
2020/21	6	7	1.17
Total	27	25	1.02

Out of 25 reconstructed males, 19 had offspring in only one season, 5 had offspring in 2 seasons, and 1 had offspring in three consecutive seasons. The breeding sex ratio ranged from 0.88 to 1.33 males per female, averaging 1.02 over the period. However, the small sample size did not allow for thorough investigation of temporal variations between nesting seasons.

### 3.5 Multiple Paternity

**FIGURE 7:**
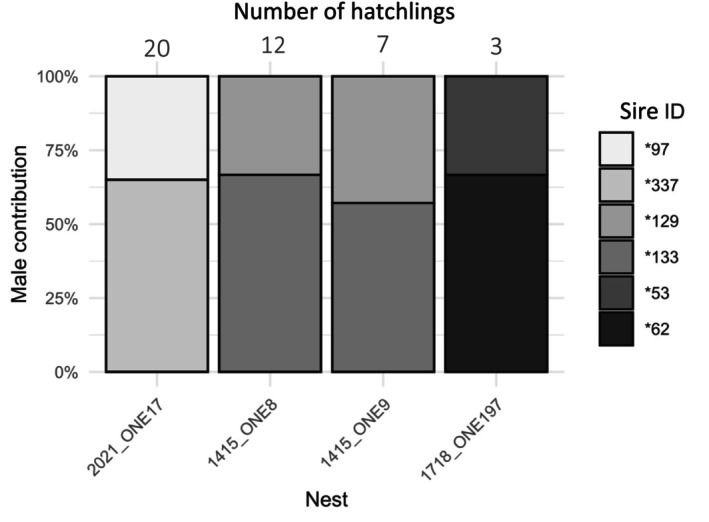
Relative sires' contribution in the four clutches where multiple paternity was found. Each vertical bar represents one clutch and each shade of grey represents one male. The number of hatchlings in the clutches is displayed above each bar.

Multiple paternity was confirmed from genotype analysis in 4 of the 37 clutches in which we identified more than two genotyped hatchlings (11%). Multiple paternity was observed in clutches containing 3–20 genotyped hatchlings, and only two sires were responsible for all sampled offspring in each clutch. One male was dominant in each clutch and sired between 60% and 66% of the genotyped hatchlings (Figure 7).

Out of these four clutches, two were laid by the same female, CMY0188 (1415_ONE8 and 1415_ONE9) (Figure 7). She laid these two clutches within 13 days in season 2014/15. They were sired by the same sires *129 and *133, with the contribution of sire *133 slightly decreasing in the second clutch (66%–60%). In this season, eight other clutches laid by the same female were sired only by sire *133, showing no multiple paternity (Table 4). Three of them were laid before the clutches with multiple paternity and numbered 3, 17 and 6 genotyped hatchlings, respectively. The remaining 5 were laid after 1415_ONE9 and numbered 8, 9, 7, 1 and 6 hatchlings (Table 4). Hatchlings from sire *129 thus were found only after several egg‐laying events.

The clutch 1718_ONE197 was laid by the female CMY1384 and sired by two sires (*62, *53) (Figure 7). For this female, we found three nests in 2017/18 counting 1, 3 and 2 genotyped hatchlings, and multiple paternity was found only in the second clutch, with sire *53 absent from the other clutches (Table 4). Finally, the last clutch with multiple paternity, 2021_ONE17, was laid by female CMY3468 in season 2020/21 and sired by sires *97 and *337. Another clutch was laid by the same female after 36 days, from which two hatchlings were sampled. Only sire *97 was found responsible for the genotype of these hatchlings (Table 4). Thus, some clutches laid by a female within a season showed multiple paternity, while others did not.

We then investigated the effect of multiple paternity on reproductive success. When comparing the indicators of reproductive success in clutches with and without multiple paternity, no significant difference was found between these clutches (Wilcoxon rank sum test *p*_values: 0.70, and 0.93 for the fertilisation success and the hatching success, respectively. Figures in Data S8).

### 3.6 Mating Behaviour and Relatedness

**FIGURE 8:**
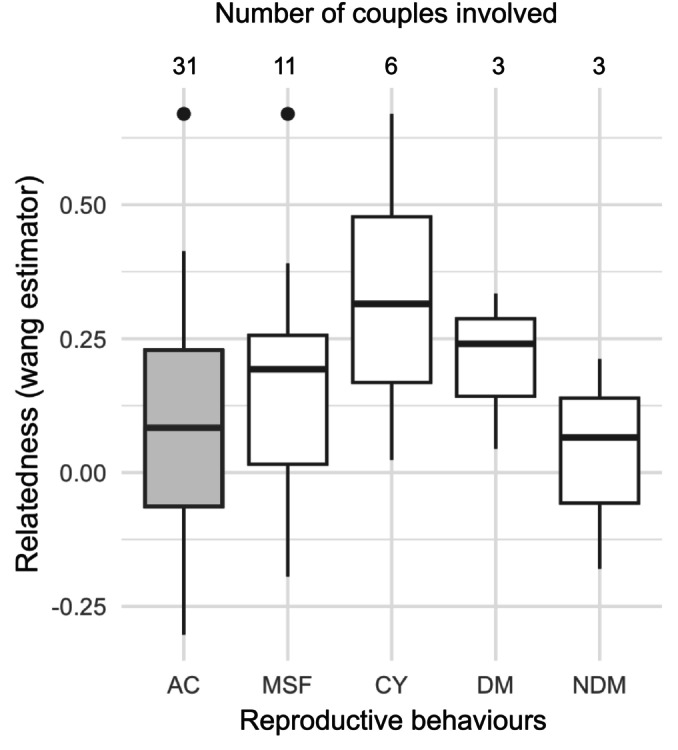
Boxplot showing median and upper/lower 25% percentile of relatedness levels in all reconstructed couples (grey boxplot) and in couples involved in several reproductive behaviours. AC: all reconstructed couples; MSF: males that mated with several females; CY: couples found in consecutive years; (N)DM: female and the (non‐)dominant sire in clutches with multiple paternity. Whiskers represent a 1.5 interquartile range, and values beyond this limit are plotted as outliers. The number of couples involved in each behaviour is shown above each boxplot.

Finally, none of the deduced mating behaviours ‐ mating with multiple partners, breeding more frequently or having a higher share of paternity in multiple paternity clutches ‐ were correlated with a change in the relatedness compared to the relatedness of all reconstructed couples (Figure 8). *T*‐test *p*‐values on the mean relatedness ranged from 0.09 to 0.66 (Table 3). However, sires that were dominant in multiple paternity clutches were consistently more related to the female than to the other sire, although that comparison involved only 6 pairs and the difference was not significant with the Wilcoxon rank sum test (*p*‐value: 0.400, Table 3). Relatedness of couples with the dominant sire ranged between 0.044 and 0.334 (median *r* = 0.241), while relatedness with the non‐dominant sire ranged between −0.180 and 0.212 (median *r* = 0.066).

**TABLE 3:** Median relatedness between potential and reconstructed couples, and couples involved in different mating behaviours.

	Median *r*	Wp	# Pairs	*r*_0.25_	*r*_0.5_
All potential couples	−0.007	—	2736	4%	0.18%
All reconstructed couples	0.084	**0.008 (vs. potential)**	31	19%	3%
Couples found in consecutive years	0.315	0.09 (vs. reconstructed)	4	—	—
Couples with multiple paternity	0.139	0.66 (vs. reconstructed)	6	—	—
Dominant male in clutches	0.240	0.29 (vs. reconstructed)	3	—	—
Non dominant male in clutches	0.066	0.40 (vs. dominant)	3		
Males mating multiple times	0.192	0.43 (vs. reconstructed)	11	—	—

*Note:* # Pairs: number of couples involved. Significant *p*‐values are indicated in bold. *r*_0.25_ (*r*_0.5_): percentage of pairs with a relatedness > 0.25 (0.5).

Abbreviation: Wp: Wilcoxon rank sum test *p*‐values.

## 4 Discussion

The present parentage analysis on the green turtle nesting population of Tetiaroa allowed for the genotypes of 27 females and 25 males to be reconstructed from the genotypes of 420 hatchlings from 105 nests, leading to a breeding sex ratio of 1.02. The highly conservative thresholds we adopted in the analysis process accounted for the small number of males and females retained, which should be interpreted as a minimal number of successful breeders rather than the ‘real’ number. As a principle, we favoured type II errors, assuming that reconstructed males and females are real and provide certainty in the following analysis. The reconstruction of male genotypes with a high degree of certainty was significantly impeded by the limited number of sampled hatchlings (fewer than 3) in the majority of the nests, which restricts the conclusions that can be drawn from this research. As a result, most of the findings discussed here should be viewed as preliminary. Nonetheless, interesting patterns related to the mating system of this green turtle population have emerged and provide directions for further research. First, the relatedness of couples was higher than expected by random mating, coupled with significant *F*_*is*_, regardless of the mating behaviour considered. Higher relatedness values were weakly correlated with higher fertilisation success in the clutches. Second, a low level of multiple paternity was observed, and individuals showed plasticity in their mating behaviours. Notably, both males and females appeared able to reproduce annually. Within a single season, females could lay several clutches over short time intervals, and males could mate with several females, both before and during the nesting season. All of these outcomes are quite new in the depiction of green turtles reproductive behaviour and features.

### 4.1 Level of Inbreeding and Strategies to Avoid Relatedness in the Population

Inbreeding was significant in this population (*F*_*is*_ = 0.06, *p*‐value < 0.001) and coupled with a higher relatedness than expected by chance. This indicates that these turtles breed non‐randomly and overall do not avoid inbreeding. A similar situation was found by Horne et al. (2022) in a Hawaiian hawksbill turtle population and was correlated with strong philopatry to nesting complexes < 100 km apart. Here, the atoll of Tetiaroa has a total area of only 6 sq. km, but each female was found nesting on only one or two of the 11 islets surveyed (Data S7). Such a level of male philopatry (< 1–2 km) would be surprising given the scale, the absence of a swimming barrier around the island, and the capacity of males to connect rookeries (Bradshaw et al. 2018; Roberts, Schwartz, and Karl 2004).

The higher levels of relatedness were correlated with higher fertilisation success, but not with hatching success. However, the correlation coefficient was low (*R* ^2^ = 0.0468), which suggests that other factors may play a role in explaining the variations in fertilisation success. Phillips et al. (2017) suggested that relatedness levels and parental multilocus heterozygosity can interact in both negative and positive ways in turtle populations and that relatedness alone does not explain the levels of reproductive success, but investigating these interactions is beyond the scope of this study. Hatching success is also influenced by other factors such as environmental conditions, which may mask the effects of relatedness and heterozygosity (Spencer and Janzen 2011).

None of the mating behaviour inferred from the parentage analysis (such as mating with multiple partners, breeding more frequently, or having a higher share of paternity in clutches with multiple sires) appeared to reduce inbreeding. The relatedness of pairs engaging in these behaviours was similar to the overall relatedness of the adult breeding population. This contradicts the inbreeding avoidance theory, which suggests that populations would generally avoid inbreeding with pre and post‐copulatory mechanisms (Blouin and Blouin 1988; Cornell and Tregenza 2007; but see Szulkin et al. 2013). Inbreeding avoidance is suspected in a small population of leatherback turtles in the South West Atlantic (Vargas et al. 2022). In contrast, the mating choices in the Tetiaroa population seem to be influenced by other factors. At the scale of French Polynesia, three distinct genetic groups were identified when analysing female and hatchling genotypes, regardless of their nesting islands and mitochondrial lineage. In Tetiaroa, these three groups coexist, and including reconstructed male genotypes in the analysis revealed that most males clustered in the same genetic group as their female mates (Data S9). This suggests a deliberate preference for partners that are genetically related rather than random pairing. Following discussions with local managers, we hypothesise that these genetic groups may be associated with phenotypical or ecotypical preferences in partner choice (e.g., carapace colour and shape) or may be induced by philopatry to breeding grounds that occur irrespective of nesting islands. In green sea turtles, mating is often believed to occur near nesting beaches (Hays, Shimada, and Schofield 2022), but it has also been observed at regional courtship areas distant from nesting beaches (Limpus 1993). In Tetiaroa, breeding behaviours are observed in the vicinity of the island (Gaspar, pers. comm.), and using satellite tracking on breeding pairs would help determine their subsequent migrations and nesting behaviour. Courtship is usually observed as a result of repetitive attempts by males, as females can also refuse to mate (Booth and Peters 1972). Green turtle courting behaviour should thus be investigated to further determine what may drive such a non‐random mating choice.

Additionally, our preliminary results on four multiple paternity clutches contradict the ‘gene compatibility’ theory, as the dominant sire in multiple paternity clutches was more related to the female than the other male. Dominance in shares of multiple paternity is thought to be linked with sperm competition (Phillips et al. 2013) and possibly result from first‐male sperm precedence (Fitzsimmons 1998; Sakaoka et al. 2011). This, for example, is confirmed in nests 1415_ONE8 and 1415_ONE9. In these nests, the dominant male was found in all of the other nests earlier in the season (Table 4), while the non‐dominant male was not found in any of the nests. Although the latter might have been missed in the early nests, this pattern may also reflect a second mating occurring later in the nesting season. Mating events after the first nest were previously found in green turtle clutches in Mexico (Chassin‐Noria et al. 2017) and in loggerhead clutches in Florida (Lasala, Hughes, and Wyneken 2020). In this case, multiple paternity might mitigate inbreeding by mating with other partners when the first one has a high level of relatedness. This mechanism is observed in the Whites' skink (*Egernia whitii*), where females sometimes seek a second and less related partner outside their social group, which increases the offspring's heterozygosity (While et al. 2014). In Chinese alligators ( *Alligator sinensis* ), polyandrous females mate with males that are less related to them than monogamous females, mitigating inbreeding in the population (Wang et al. 2017). To date, very few studies have explored relatedness in multiple paternity clutches of marine turtles (Phillips et al. 2013). This hypothesis therefore needs to be confirmed with a higher number of multiple paternity clutches and deserves further investigations in other populations.

**TABLE 4:** Sire ID and number (nb) of genotyped offspring in all clutches of the three females involved in polyandry.

Female ID	CMY0188		CMY1384		CMY3468	
Season	2014/15		2017/18		2020/21	
Nest number	Sire ID	Offspring nb	Sire ID	Offspring nb	Sire ID	Offspring nb
#1	*133	3	*62	1	*97 ‐ *337	**20 (7–13)**
#2	*133	17	*62 ‐ *53	**3 (2–1)**	*97	2
#3	*133	6	*62	2		
#4	*133 ‐ *129	**12 (8–4)**				
#5	*133 ‐ *129	**7 (4–3)**				
#6	*133	8				
#7	*133	9				
#8	*133	7				
#9	*133	1				
#10	*133	6				

*Note:* Clutches with multiple paternity are indicated in bold, and the number of genotyped offspring for each sire is indicated in brackets.

### 4.2 Number of Breeders and Population Density

The average breeding sex ratio over the years (BSR) was close to one (1.02:1 male: female), indicating that approximately an equal number of males and females were breeding in this population. Breeding and OSRs in sea turtles have typically been reported as skewed towards males or even between both sexes (Hays, Shimada, and Schofield 2022). For example, Prakash et al. (2022) found a BSR of 1:1 in a population of hawksbill turtles in Fiji, and Wright, Stokes, et al. (2012) found an OSR of 1.4:1 in a green turtle population in Cyprus. The primary sex ratio of this population in Tetiaroa does not seem to suffer heavy female bias as observed elsewhere (Laloë et al. 2020), and so the discrepancy between the number of male and female breeders is likely small. However, a low level of multiple paternity was observed (11% of the clutches) and whereas other green turtle populations demonstrated multiple paternity in as much as 92% of their clutches (Alfaro‐Núñez, Jensen, and Abreu‐Grobois 2015). Our finding should be considered as a conservative estimate of the level of multiple paternity in this population, as only 37 nests (out of 105 analysed) had more than 3 sampled hatchlings and only 6 nests had more than 10 hatchlings. The contribution of some sires has likely been overlooked, and the actual level of multiple paternity in this population is likely higher. With no apparent benefits on the indicators of reproductive success, a low level of multiple paternity may indicate low chances of encountering multiple males, directly linked to the density and number of male breeders (Lee et al. 2018). However, a high density of breeders is observed near the island (Gaspar, pers. comm.), and 11% of the males mated with multiple females within a season, indicating that males may encounter several available females. Coupled with the low level of multiple paternity, it suggests that not all males are equally capable of mating with multiple females. In contrast, Phillips et al. (2013) did not find any male hawksbills that mated multiple times within a season and concluded that a low density and high turnover of males prevented this from occurring.

These results represent the first investigation of the number of male breeders and the level of multiple paternity in French Polynesia. They indicate that there is a sufficient number of males to preserve the population's reproductive success, despite the high level of relatedness.

### 4.3 Plasticity in Mating Behaviours

#### 4.3.1 Reproduction Frequency and Long‐Term Sperm Storage

Parentage assignment revealed that at least five females (18%) returned to lay once or twice during the 11‐year study period. These returning events were not observed in field surveys, as recapture identification found that only two females were resampled during a returning event. One of these females was among the five females identified from their genotype, matching the genetic approach (CMY1113, Table 1). Interestingly, four of these females were found nesting in consecutive seasons. To the best of our knowledge, this represents the first record of annual remigration interval for female green turtles in their natural environment. Studies focusing on female reproduction frequency are based on field observations, and thus it is possible that in some cases, annual reproduction has been overlooked due to the absence of direct observation in the field.

Satellite tracking on adult females in French Polynesia revealed that several individuals stay within the territory, while others migrate to Fijian neritic feeding grounds between the nesting seasons (Craig et al. 2004; Piovano et al. 2019; DIREN personal comm.). Whether those breeding annually are those that remain in French Polynesia throughout the year needs to be determined, but it is difficult to imagine long annual migrations to Fiji (> 2000 km), given the time and energy that it requires, adding to the energy needed for the reproduction itself. Interestingly, the same sires consistently sired the clutches of annually breeding females over the given period (from 359 to 673 days). This may indicate that females are capable of storing sperm across seasons or exhibiting narrow male choice for reproduction, implying fidelity to what are currently unknown mating criteria. Alternatively, cross‐seasonal sperm storage has neither been demonstrated nor quantified in marine turtles, although it is suspected to occur. For example, Wright et al. (2013) found that multiple paternity was more common in returning females than in primary nesters, and proposed cross‐seasonal sperm storage as one of the possible causes. Cross‐seasonal sperm storage over several years has been observed in other taxa, including birds (Feldschuh et al. 2005), insects (Baer et al. 2003), and reptiles (Ewing 1943; Levine, Schuett, and Booth 2021). Although the possibility of multiple mating among the same individuals cannot be ruled out (Sakaoka et al. 2013), cross‐seasonal sperm storage coupled with relatively sedentary life traits may help females minimise the energy costs linked with migration and mating. To date, however, adult green turtles are rarely observed outside the nesting season in French Polynesia, and no feeding ground has been identified throughout French Polynesia or nearby. Hatase et al. (2006) showed, using satellite tracking and stable isotope analysis, that 31% (*n* = 89) of the females nesting on Ogasawara Islands, Japan, were oceanic planktivores rather than neritic herbivores. Further investigation using these complementary techniques should reveal whether females in French Polynesia also feed in oceanic habitats close to nesting grounds.

Regarding the males, 4 (16%) sired different females in consecutive seasons, indicating a possible annual reproduction frequency. However, caution should be taken as this might also reflect long‐term sperm storage in the case when the females could not be identified in one of the consecutive seasons. Male reproduction interval is thought to be shorter than for females due to a smaller energy cost of reproduction that allows them to reproduce more often (Hays, Shimada, and Schofield 2022). For example, the male green turtle remigration interval was 2.1 years in the southern Great Barrier Reef (Limpus 1993), and 1 year in Hawaii (Balazs 1983), based on tagged recapture. Wright, Fuller, et al. (2012) determined the reproduction frequency of male green turtles in northern Cyprus with parentage analysis and found that 3% bred more than once within the 3 years. Overall, annual male reproduction frequency linked to a residential strategy must be considered as a strong possibility in French Polynesia, and in this population, a discrepancy between male and female reproduction frequency is not observed.

#### 4.3.2 Within‐Season Nesting Parameters

Within a single season, females laid on average 3.2 clutches. Field estimations based on the length of the nesting season and the observed inter‐nesting interval proposed that each female laid on average 6 nests in 2017/18 (Touron, Genet, and Gaspar 2018). The true number is likely to lie between these two estimations and is coherent with clutch frequencies for other green turtle populations in the Pacific Ocean (reviewed in Pilcher 2021). They vary from 1.8 in French Frigate Shoal to 7 ± 1.3 clutches per female per season in the Northern Mariana Islands (Balazs 1980; Summers et al. 2018). Additionally, through parentage analysis, 74 nests without observed females in the field were associated with a female, demonstrating the efficiency of the analysis to supplement field observations.

Inter‐nesting intervals were highly variable (between 0 and 94 days). The extended intervals likely suggest missed nesting events due to the inability to analyse all nests. Alternatively, the females may be nesting in other locations during the season, although sightings of nesting and hatching events are rare on the nearest islands (Tahiti, Moorea, > 55 km) and the habitat is less suitable due to a more artificialized coastline (DIREN, pers.com.). An interval of 11–14 days was the most common in this population. This is coherent with field observations (Touron, Genet, and Gaspar 2018), and aligns with findings in other populations (reviewed in Robinson et al. 2022). However, and more surprisingly, our study reveals the ability of females to lay regular‐sized clutches within a very short interval (0–3 days). If confirmed with a larger data set and physiological data, this would represent a new finding in the reproductive strategy of green turtles. This would show a capacity for plasticity in nesting behaviour, a potential advantage in allowing a population to adapt to changing environmental and weather conditions on nesting beaches if one supposes that females might search for optimal nesting conditions. Plasticity in nesting behaviour has been observed in Tetiaroa, with an increase in the nesting season length over the years (Touron, Genet, and Gaspar 2018), possibly indicating that females may look to nest during cooler months to produce more male offspring under the ongoing warmer conditions (Laloë et al. 2020).

For almost all females, clutches were sired by the same sire within a season, coherent with the fact that breeding occurs before nesting (Hays, Shimada, and Schofield 2022). However, one male (*129) sired 2 clutches in the middle of the nesting season, which induced multiple paternity in these clutches. The genes of this male were not found in the first three clutches nor the last five clutches laid by the same female (CMY0188) during the season, although the number of sampled hatchlings (1–17) was enough to detect multiple paternity in some of these clutches. The first clutch sired by this male was laid 40 days after the first recorded clutch for this female. Female marine turtles are thought to be available for reproduction only for a short interval of approximately 1 week, before nesting (Comuzzie and Owens 1990). Here we suggest that mating can also occur during the inter‐nesting interval (see Chassin‐Noria et al. 2017 and Lasala, Hughes, and Wyneken 2020 for additional examples), which indicates that green turtles are capable of unexpected plasticity in their mating behaviour.

### 4.4 Implications for Conservation

The green turtle population of Tetiaroa shows a significant level of relatedness and inbreeding, with no clear inbreeding avoidance strategy and an apparent preference for related partners that rather favours it. This indicates that the reproductive system of green turtles makes them intrinsically vulnerable to inbreeding and its potential negative consequences on the capacity of a population to maintain itself through generations. Although it does not seem to affect the reproductive success of Tetiaroa's population at this stage, it could have negative effects if the number of breeders is not maintained, which highlights the importance of turtle conservation programmes. On Tetiaroa, a conservation and monitoring programme has been in place since 2007, and an increasing number of breeders has been recorded on the island, indicating that it is likely efficient and can preserve the genetic diversity of this population (Touron, Genet, and Gaspar 2018). Finally, unexpected plasticity in green turtle mating behaviours and nesting parameters shows some adaptive capacity which may help maintain the population's resilience under changing environmental conditions.

## Author Contributions

**Violaine Dolfo:** conceptualization (supporting), data curation (lead), formal analysis (lead), investigation (lead), methodology (lead), writing – original draft (lead). **Cécile Gaspar:** data curation (supporting), resources (lead), writing – review and editing (supporting). **Miri Tatarata:** funding acquisition (equal), project administration (equal). **Emilie Boissin:** conceptualization (supporting), data curation (supporting), formal analysis (supporting), methodology (supporting), supervision (equal), validation (equal), writing – review and editing (equal). **Serge Planes:** conceptualization (lead), data curation (supporting), formal analysis (supporting), funding acquisition (equal), methodology (supporting), project administration (equal), supervision (equal), validation (equal), writing – review and editing (equal).

## Ethics Statement

*Chelonia mydas* sample collection was authorised and coordinated by the Direction of the Environment of French Polynesia. Non‐lethal skin and muscle biopsies were performed, which do not require any other specific permits. Samples were exported to France for processing with CITES permits n° FR1298700118‐E and n° FR2098700187‐E. All samples remain the DIREN's property.

## Conflicts of Interest

The authors declare no conflicts of interest.

## Supporting information


Data S1.

Data S2.

## Data Availability

Microsatellite sequences are available on GenBank under accession number OQ162049‐OQ162073. All other data generated or analysed during this study are included in this published article and its Supporting Information.
