# Supplementary material for: Parentage Analysis in a Green Sea Turtle (Chelonia mydas) Population From French Polynesia Reveals a Tendency for Inbreeding and Unexpected Plasticity in Reproductive Behaviour
Source: Ecol Evol. 2025 Jul 16;15(7):e70855. doi: 10.1002/ece3.70855 (PMC12264388; doi:10.1002/ece3.70855)
Supplement: Supplementary file 2 — Data S2. [file ECE3-15-e70855-s002.docx]

**Supplemental Information for:**

**Parentage analysis in green sea turtle (*Chelonia mydas*) population from French Polynesia reveals a tendency for inbreeding and unexpected plasticity in the reproductive behaviours**

Violaine Dolfo, Cécile Gaspar, Miri Tatarata, Emilie Boissin, Serge Planes

**Table of Contents:**

| **Supplementary data S2** | Page 2 |
| --- | --- |
| **Supplementary data S4** | Page 4 |
| **Supplementary data S6** | Page 5 |
| **Supplementary data S8** | Page 5 |
| **Supplementary data S9** | Page 6 |

**Supplementary data S2**. COLONY Calibration with different sets of microsatellite markers


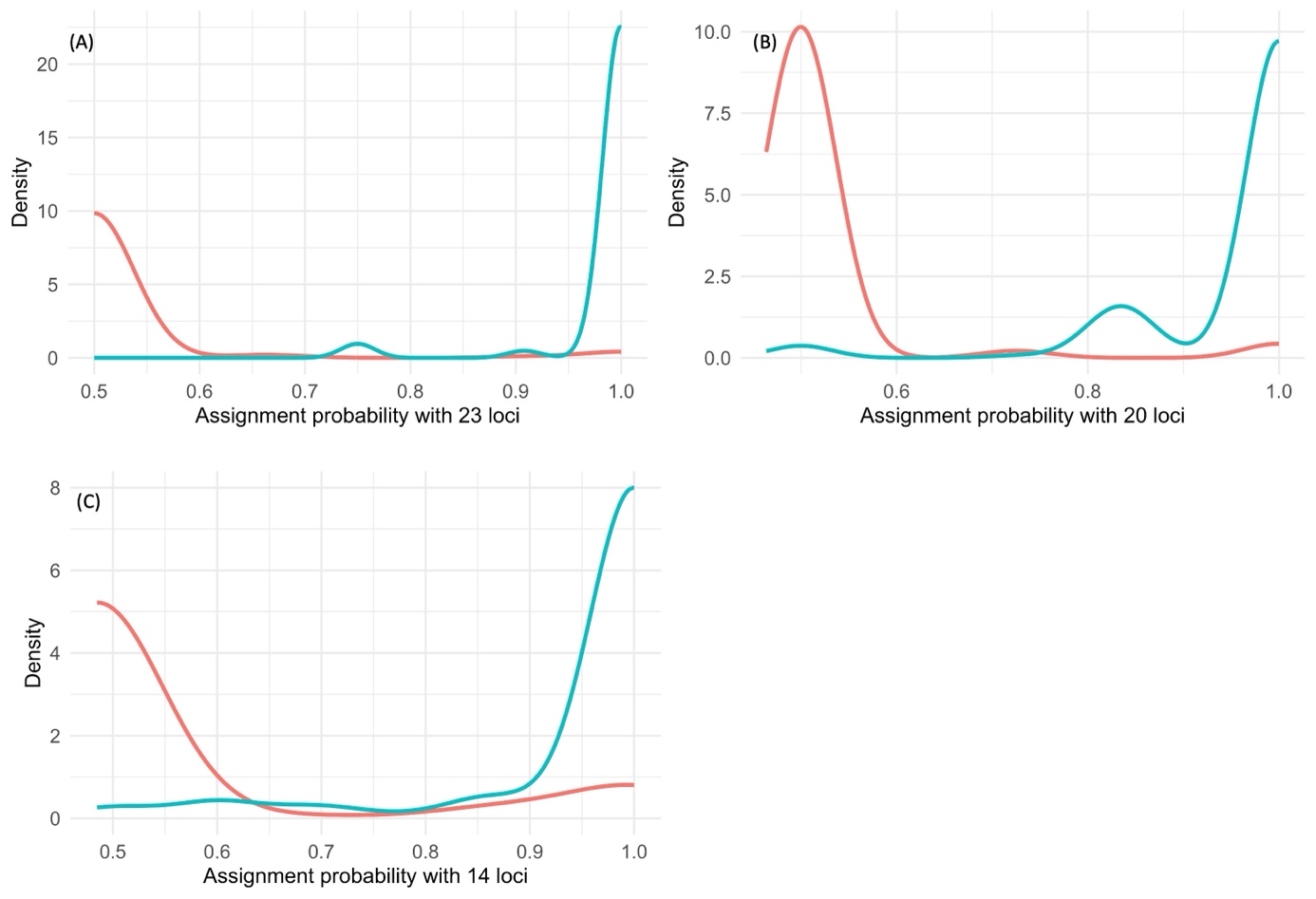


Figure S2. Density of mother-offspring COLONY assignment probabilities for the known mother-offspring relationships of our dataset, and different sets of loci. This calibration was made with 58 mother genotypes and 275 hatchling genotypes. True motherhood is represented with the blue line, and type II errors are represented with the red line (i.e. “false” motherhood). (A) using the 23 microsatellite markers of Dolfo et al. (2023). (B) using only 20 of these markers. (C) using only the markers that do not deviate from Hardy-Weinberg equilibrium. See below the list of markers for (A), (B), (C). Retaining 23 loci (A) minimises type II errors and allows to determine a clear threshold for motherhood probability assignment.

Table S2. List of microsatellite markers used in simulations (A), (B), and (C) of Figure S1. The markers are those described in Dolfo et al. (2023), the error rate was calculated with COLONY during the simulation. In bold are indicated the markers with the highest error rates, which were removed for Simulation B, and * indicates the markers deviant from Hardy-Weinberg equilibrium and removed for Simulation C.

| Simulation (A) – 23 markers | | Simulation (B) – 20 markers | Simulation (C) - 14 markers |
| --- | --- | --- | --- |
| markers | error rate | markers | markers |
| CMY03 | 0.011 | CMY03 | CMY03 |
| CMY04 | 0.011 | CMY04 | CMY04 |
| CMY08 | 0.015 | CMY08 | CMY08 |
| CMY09* | 0.001 | CMY09 | CMY16 |
| CMY10* | 0 | CMY10 | CMY17 |
| CMY11* | 0 | CMY11 | CMY20 |
| CMY14* | 0.005 | CMY14 | CMY21 |
| CMY15* | 0.011 | CMY15 | CMY22 |
| CMY16 | 0.014 | CMY16 | CMY25 |
| CMY17 | 0.019 | CMY17 | CMY27 |
| CMY18* | 0.019 | CMY18 | CMY29 |
| **CMY19*** | **0.026** | CMY20 | CMY32 |
| CMY20 | 0.002 | CMY21 | CMY35 |
| CMY21 | 0.008 | CMY25 | CMY45 |
| **CMY22** | **0.032** | CMY26 |  |
| CMY25 | 0.010 | CMY27 |  |
| CMY26* | 0.002 | CMY29 |  |
| CMY27 | 0.009 | CMY33 |  |
| CMY29 | 0 | CMY35 |  |
| **CMY32** | **0.029** | CMY45 |  |
| CMY33* | 0.005 |  |  |
| CMY35 | 0.004 |  |  |
| CMY45 | 0.006 |  |  |

Dolfo, V., Boissin, E., Tatarata, M., Planes, S., 2023. Characterization of 25 new microsatellite markers for the green turtle (Chelonia mydas) and cross-species amplification in other marine turtle species. Mol. Biol. Rep. https://doi.org/10.1007/s11033-023-08341-4

**Supplementary data S4. Comparison between the relatedness estimators implemented in the R package *related* based on observed allele frequencies and 100 simulated genotypes in each relationship category.**

Theoretical relatedness values for each category. Full-Sibs: 0.5; Half-Sibs: 0.25; Parent-Offspring: 0.5; Unrelated:0. Estimators. L&L: Li et al. (1993); L&R: Lynch & Ritland (1999); Q&G: Queller & Goodnight (1989); W: Wang (2002).

**
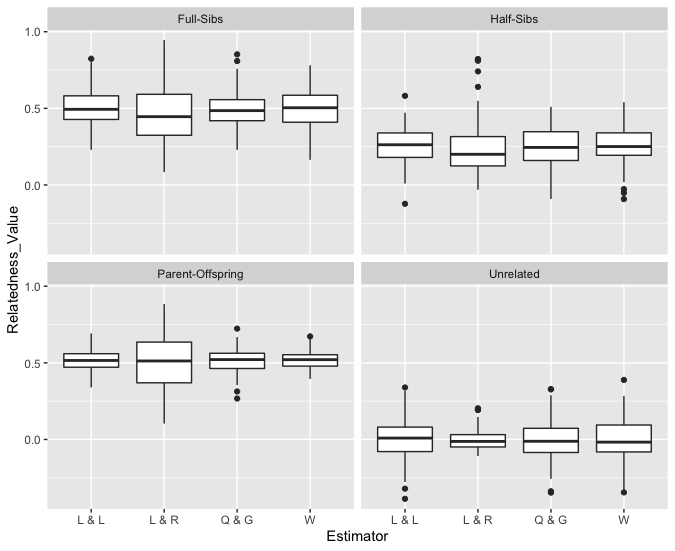
**

Correlation Coefficients between Observed & Expected values:

W 0.884378

L&L 0.882150

L&R 0.777613

Q&G 0.871067

**Supplementary data S6. Number of nests per female within a single season.**

| **Number of females** | **Number of laid nests** |
| --- | --- |
| 7 | 1 |
| 7 | 2 |
| 3 | 3 |
| 8 | 4 |
| 5 | 5 |
| 1 | 8 |
| 1 | 10 |

**Supplementary data S8. Indicators of reproductive success in clutches with and without multipaternity.**


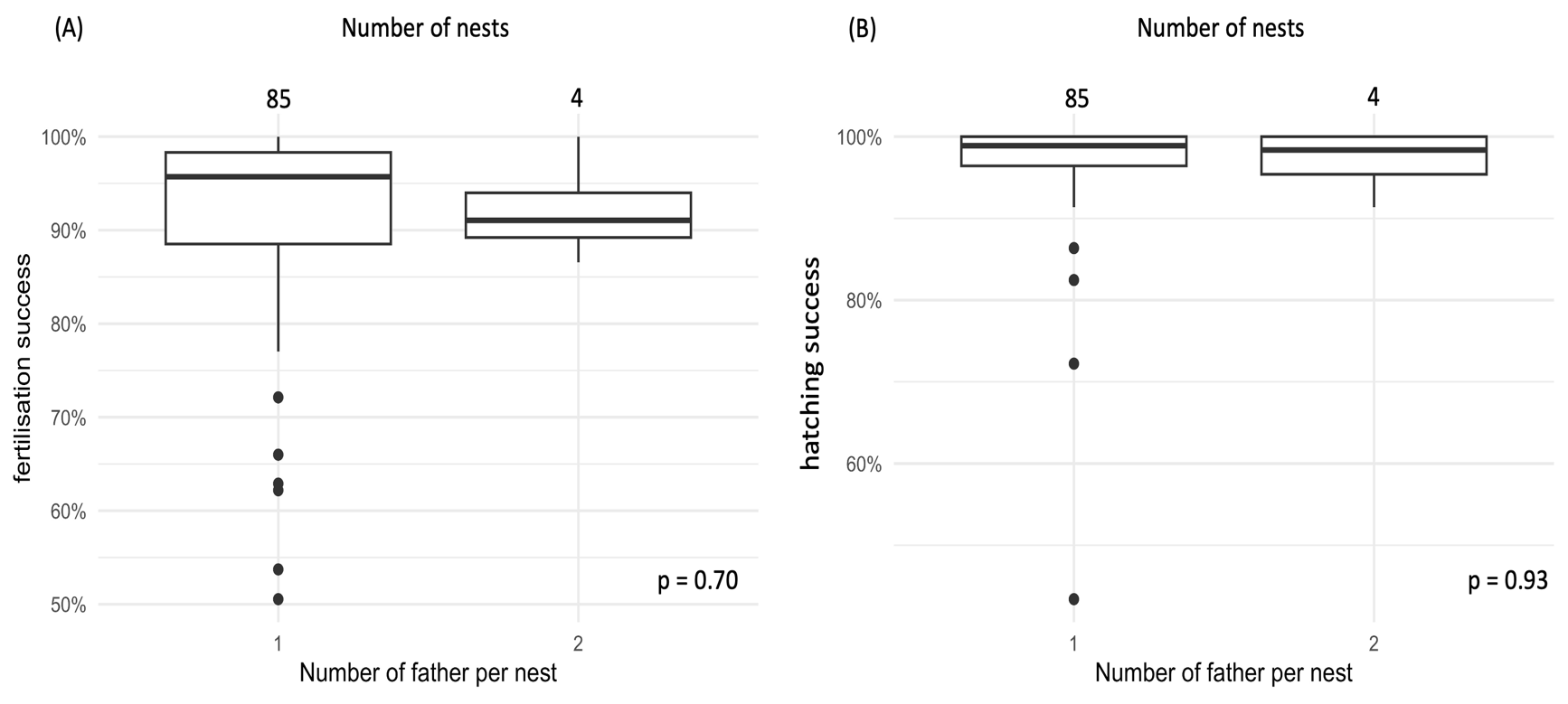


Figure S8 Boxplot showing median and upper/lower 25% percentile of fertilisation success (A), and hatching success (B) in the nests with 1 and 2 fathers. Whiskers represent a 1.5 interquartile range, and values beyond this limit are plotted as outliers. The number of nests in each group (ie. with 1 or 2 fathers) is shown above each boxplot. p: p-value of the non-parametric Wilcoxon Rank Sum test on the median.

**
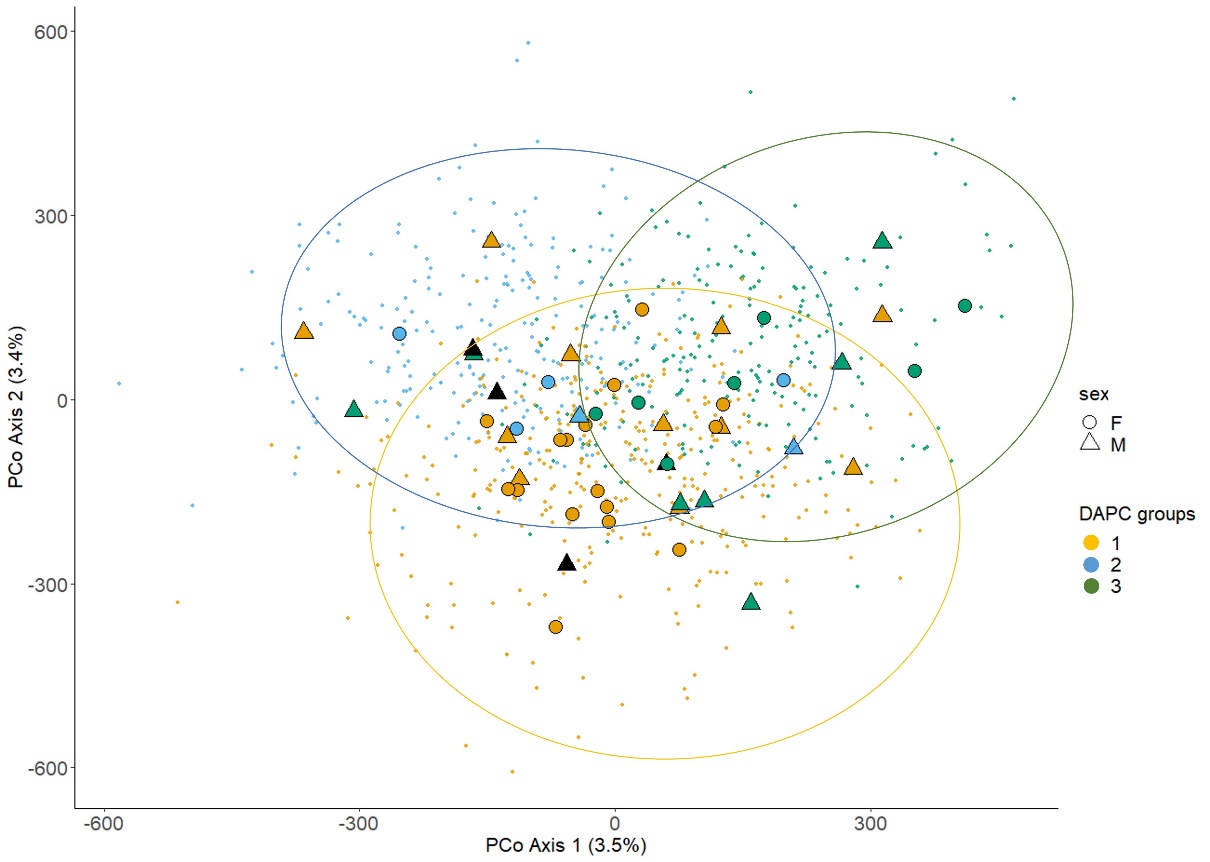
Supplementary data S9. Projection of females and reconstructed males of Tetiaroa in the PCoA showing three populations in French Polynesia (Dolfo et al., in prep)**

Figure S9. Reconstructed males are indicated with triangles, females are shown with circles. The individuals used in this PCoA are indicated with small dots and cluster into three groups, materialised with circles and different colours. PCoA groups are those found by Dolfo et al. (in prep). The females emphasised here were included in the initial PCoA analysis. Reconstructed males were assigned the group in which the female they mated with was found. Black triangles represented males that mated with females belonging to group 1 and group 2.
